# Contribution of clinical information to the predictive performance of plasma β-amyloid levels for amyloid positron emission tomography positivity

**DOI:** 10.3389/fnagi.2023.1126799

**Published:** 2023-03-14

**Authors:** Min Young Chun, Hyemin Jang, Hee Jin Kim, Jun Pyo Kim, John Gallacher, José Antonio Allué, Leticia Sarasa, Sergio Castillo, María Pascual-Lucas, Duk L. Na, Sang Won Seo, on behalf of DPUK

**Affiliations:** ^1^Departments of Neurology, Samsung Medical Center, Sungkyunkwan University School of Medicine, Seoul, Republic of Korea; ^2^Department of Neurology, Yonsei University College of Medicine, Seoul, Republic of Korea; ^3^Department of Neurology, Yongin Severance Hospital, Yonsei University Health System, Yongin, Republic of Korea; ^4^Neuroscience Center, Samsung Medical Center, Seoul, Republic of Korea; ^5^Alzheimer's Disease Convergence Research Center, Samsung Medical Center, Seoul, Republic of Korea; ^6^Department of Health Sciences and Technology, SAIHST, Sungkyunkwan University, Seoul, Republic of Korea; ^7^Department of Digital Health, SAIHST, Sungkyunkwan University, Seoul, Republic of Korea; ^8^Center for Neuroimaging, Radiology and Imaging Sciences, Indiana University School of Medicine, Indianapolis, IN, United States; ^9^Department of Psychiatry, Warneford Hospital, University of Oxford, Oxford, United Kingdom; ^10^Araclon Biotech-Grifols, Zaragoza, Spain; ^11^Department of Clinical Research Design and Evaluation, SAIHST, Sungkyunkwan University, Seoul, Republic of Korea

**Keywords:** Alzheimer’s disease, β-Amyloid, positron emission tomography, cerebrospinal fluid, plasma, apolipoprotein E

## Abstract

**Background:**

Early detection of β-amyloid (Aβ) accumulation, a major biomarker for Alzheimer’s disease (AD), has become important. As fluid biomarkers, the accuracy of cerebrospinal fluid (CSF) Aβ for predicting Aβ deposition on positron emission tomography (PET) has been extensively studied, and the development of plasma Aβ is beginning to receive increased attention recently. In the present study, we aimed to determine whether *APOE* genotypes, age, and cognitive status increase the predictive performance of plasma Aβ and CSF Aβ levels for Aβ PET positivity.

**Methods:**

We recruited 488 participants who underwent both plasma Aβ and Aβ PET studies (Cohort 1) and 217 participants who underwent both cerebrospinal fluid (CSF) Aβ and Aβ PET studies (Cohort 2). Plasma and CSF samples were analyzed using ABtest-MS, an antibody-free liquid chromatography-differential mobility spectrometry-triple quadrupole mass spectrometry method and INNOTEST enzyme-linked immunosorbent assay kits, respectively. To evaluate the predictive performance of plasma Aβ and CSF Aβ, respectively, logistic regression and receiver operating characteristic analyses were performed.

**Results:**

When predicting Aβ PET status, both plasma Aβ42/40 ratio and CSF Aβ42 showed high accuracy (plasma Aβ area under the curve (AUC) 0.814; CSF Aβ AUC 0.848). In the plasma Aβ models, the AUC values were higher than plasma Aβ alone model, when the models were combined with either cognitive stage (*p* < 0.001) or *APOE* genotype (*p* = 0.011). On the other hand, there was no difference between the CSF Aβ models, when these variables were added.

**Conclusion:**

Plasma Aβ might be a useful predictor of Aβ deposition on PET status as much as CSF Aβ, particularly when considered with clinical information such as *APOE* genotype and cognitive stag*e*.

## Introduction

Alzheimer’s disease (AD), one of the most common neurodegenerative diseases, is caused by abnormal deposition of β-amyloid (Aβ) in the brain (Jack et al., 2018). Early diagnosis of AD has been possible through the development of Aβ positron emission tomography (PET) (Albert et al., 2011; McKhann et al., 2011; Dubois et al., 2014). As an important fluid biomarker, cerebrospinal fluid (CSF) Aβ has also been known to reflect neuropathological processes of Aβ deposition through autopsy studies (Strozyk et al., 2003; Engelborghs et al., 2008) with a high accuracy of 86.6% (Tapiola et al., 2009). In addition, previous studies showed the high concordance of 84–92% between Aβ PET and CSF Aβ (Zwan et al., 2014; Jack et al., 2018; Lee et al., 2020). However, some discordances between Aβ PET and CSF Aβ were also reported (Jung et al., 2020; Sala et al., 2021), suggesting that CSF Aβ reflects earlier Aβ changes than PET in the brain, or alternatively, that CSF Aβ and Aβ PET might represent different pathophysiology including spatial tau patterns (Jang et al., 2021).

In recent years, plasma Aβ biomarkers are receiving increased attention as another promising fluid biomarkers since they might overcome the limitations of PET or CSF biomarkers in terms of difficult access to equipment, high cost (Johnson et al., 2013), or invasiveness. Some studies have also suggested that plasma Aβ could predict Aβ PET status (Fandos et al., 2017; Nakamura et al., 2018; Schindler et al., 2019; Jang et al., 2021; Benedet et al., 2022; Li et al., 2022). However, other studies have shown that the concordance between plasma Aβ and Aβ PET was 75.5–80.8% (Meyer et al., 2022), which is lower than that between CSF Aβ and Aβ PET. Moreover, it is unknown whether plasma Aβ reflects post-mortem Aβ plaques as much as CSF Aβ. Thus, the biological variability of plasma Aβ biomarkers for predicting Aβ deposition on PET should be further investigated.

Cerebrospinal fluid (CSF) Aβ and plasma Aβ have distinctive characteristics and may represent different pathogenic mechanisms. That is, Aβ in the brain is removed by variety of mechanisms including transportation across the blood–brain barrier (BBB) (Monro et al., 2002; Roberts et al., 2014) into the venous blood and reabsorption into the venous circulation *via* CSF (Roberts et al., 2014). Therefore, factors affecting BBB might have an influence on plasma Aβ levels. There are several factors affecting permeability and transport across the BBB including *APOE* genotypes, age, and cognitive stage (Jack et al., 2018). Thus, we hypothesized that *APOE* genotypes, age, and cognitive status might affect the predictive performance of plasma Aβ, but not CSF Aβ levels for Aβ uptakes on PET.

In the present study, we aimed to determine whether *APOE* genotypes, age, and cognitive stage affect the predictive performance of fluid Aβ levels for amyloid PET positivity in Aβ plasma—Aβ PET cohort (Cohort 1) and Aβ CSF—Aβ PET cohort (Cohort 2).

## Materials and methods

### Study participants

#### Cohort 1: Aβ Plasma—Aβ Pet cohort

We searched the Korea-Registries to Overcome and Accelerate Dementia research project (K-ROAD) database for participants who underwent both Aβ PET and Aβ plasma studies. The K-ROAD aims to develop a genotype–phenotype cohort to accelerate the development of novel diagnostic and therapeutic techniques for Alzheimer’s and concomitant cerebrovascular disease. Nation-wide, 25 university-affiliated hospitals in South Korea are participating in the K-ROAD. This strategy identified a consecutive series of 488 participants. The syndromal staging of cognitive continuum included cognitively unimpaired (CU), those with amnestic mild cognitive impairment (aMCI), or those with AD dementia (ADD) who were diagnosed by the National Institute on Aging—Alzheimer’s Association (NIA–AA) Research Framework (Jack et al., 2018). We combined participants with aMCI and ADD to build up the cognitive impaired (CI) group.

All participants were assessed through clinical interviews and neurological examinations, and clinical diagnoses were established by consensus among a multidisciplinary team. Blood tests included complete blood count, blood chemistry, vitamin B12/folate measurement, syphilis serology, thyroid function test, and *APOE* genotyping. They also underwent a standardized neuropsychological test [Seoul Neuropsychological Screening Battery, SNSB (Ahn et al., 2010; Kang et al., 2012)], and brain magnetic resonance imaging (MRI). Patients were excluded if they had territorial infarctions, cortical strokes, brain tumors, or vascular malformations on MRI. Patients with white matter hyperintensities due to radiation injury, multiple sclerosis, vasculitis, or leukodystrophy were also excluded.

#### Cohort 2: Aβ CSF—Aβ Pet cohort

We searched K-ROAD database for participants who underwent both Aβ PET and Aβ CSF studies. This strategy identified a consecutive series of 217 participants. They also have followed the same diagnostic process as participants within Cohort 1.

Written informed consent was obtained from the SMC in South Korea, and the institutional review board approved the study protocol.

### Amyloid pet imaging and analysis

All participants underwent either ^18^F-florbetaben (FBB) or ^18^F-flutemetamol (FMM) PET at SMC using a Discovery STe PET/computed tomography (CT) scanner (GE Medical Systems, Milwaukee, WI, United States) in 3D scanning mode that examined 47 slices of 3.3-mm thickness spanning the entire brain (Kim et al., 2018; Jang et al., 2019). CT images were acquired using a 16-slice helical CT (140 KeV, 80 mA;3.75-mm section width) for attenuation correction. According to the protocols proposed by the ligands’ manufacturers, a 20-min emission PET scan with dynamic mode (consisting of 4 × 5 min frames) was performed 90 min after injection of a mean dose of 311.5 MBq of FBB or 185 MBq of FMM. 3D PET images were reconstructed in a 128 × 128 × 48 matrix with a voxel size of 2 mm × 2 mm × 3.27 mm using the ordered-subsets expectation maximization algorithm (FBB iterations = 4 and subset = 20; FMM iterations = 4 and subset = 20).

Positron emission tomography images were co-registered on the individual 3D-T1 weighted MR images that were normalized to T1-weighted MNI-152 template using the Statistical Parametric Mapping (SPM) 8. Cerebral cortex segmentation was derived from the segmentation method on the SPM8 and Automatic anatomical labeling (AAL) template. The whole cerebellum (WC) mask was downloaded from the Global Alzheimer’s Association Interactive Network (GAAIN) website.^1^ Any corrections were not applied on PET images for brain atrophy or partial volume effects.

We replicated the image processing steps described in the previous study, direct comparison Centiloid (dcCL) study (Cho et al., 2020a), based on the Centiloid project (Klunk et al., 2015). FBB-FMM cortical target region (CTX VOI) derived SUVR was converted as the dcCL with transformation equation derived from previous studies of FBB (dcCL_FBB_ = 151.42 × dcSUVR_FBB_–142.24) and FMM (dcCL_FMM_ = 148.52 × dcSUVR_FMM_–137.09) (Cho et al., 2020a,b).

To obtain the dcCL cutoff value for Aβ PET positivity, we performed receiver operating characteristic (ROC) analysis using Aβ PET positivity based on the SUVR cutoff for each PET scan as the standard of truth. We defined Aβ PET positivity according to the cutoff value of the FBB or FMM PET global dcCL, which was previously described and computed as 25.11 (Jang et al., 2021).

### Plasma Aβ collection and processing

We obtained 8 ml of blood from each participant and placed into a 0.5 M EDTA-containing tube and mixed it for 5 min (Jang et al., 2021). The Green Cross lab picked up the samples that were stocked in the cooler after mixing. Plasma was extracted from the blood sample after a 10-min centrifuge (1,300 g) and dispensed into 5 or 10 vials at a volume of 0.3 ml each. All plasma samples were kept frozen at −75°C until LC–MS analysis. The process complied with the manual for human resource collection and registration of the National Biobank of the South Korea (Johnson et al., 2007).

### Plasma Aβ liquid chromatography-mass spectrometry (LC–MS)

The prepared plasma samples were sent to Araclon Biotech (Zaragoza, Spain) and analyzed using LC–MS (Jang et al., 2021). Plasma samples were analyzed using ABtest-MS, an antibody-free liquid chromatography-differential mobility spectrometry-triple quadrupole mass spectrometry (HPLC-DMS-MS/MS) method (Jang et al., 2021). The analytical platform was composed of a QTRAP 6500+ hybrid linear ion trap-triple quadrupole mass spectrometer fitted with a differential mobility spectrometry interface (SelexION) and coupled to an M3 Micro LC system (Sciex, Framingham, MA, United States). Samples (200 μl each) were analyzed singles. Analytes were extracted directly from plasma, and no immunoprecipitation procedure was performed. Intact Aβ40 and Aβ42 species were analyzed as no enzymatic digestion was performed. The specifics of the method are the subject matter of patent application (EP2020382352).

### Analysis of plasma Aβ mass spectrometry data

Calibration curves were prepared in human plasma after spiking ^15^N-Aβ40 and ^15^N-Aβ42 at seven concentration levels. Quality control samples were also prepared in human plasma at three concentration levels (low: 3 × LLOQ, mid, and high). The calibration ranges were 50–1,000 pg./ml for ^15^N-Aβ40 and 10–200 pg./ml for ^15^N-Aβ42. The LLOQ for ^15^N-Aβ40 was 50 pg./ml (% relative error RE = 0.3% and coefficient of variation CV = 7%). The LLOQ for ^15^N-Aβ42 was 10 pg./ml (RE = −1.5% and CV = 11%).

Two calibration curves were used in each analytical run, one at the beginning and one at the end of the sequence. Additionally, six quality control samples, uniformly distributed along the sequence, were analyzed in each run.

Deuterated internal standards (^2^H-Aβ40 and ^2^H-Aβ42) were spiked in all samples (calibration curves, quality control, and study samples). Response ratios corresponding to endogenous species in the study samples (^14^N-Aβ40/^2^H-Aβ40 and ^14^N-Aβ42/^2^H-Aβ42) were interpolated in the calibration curves made with ^15^N analogs. Suitability test samples were analyzed every day at the beginning of the analytical run to evaluate system performance and equal transmission for light (^14^N) and heavy (^15^N) species.

Analyst 1.6.3. Software (Sciex) was used for data acquisition, and the MultiQuant 3.0.3. software (Sciex) was used for data processing.

Since plasma Aβ42/Aβ40 ratio measured by the LC–MS method has previously shown good performance in discriminating Aβ PET positivity (Jang et al., 2021), we used the plasma Aβ42/Aβ40 ratio as the plasma Aβ variable.

### Cerebrospinal fluid (CSF) Aβ study and analysis

Cerebrospinal fluid samples were collected from a lumbar puncture done in the L3-4 or L4-5 intervertebral spaces using a 20 or 22G needle. Fasting was not required. All CSF samples were collected into 15-mL polypropylene tubes at the time of the tap and were then sent to Samsung Medical Center laboratory within 30 min after collection (Lee et al., 2020). After samples were centrifuged at 2000 *g* for 10 min within 4 h after collection, aliquots (1 ml) prepared from these samples at room temperature were immediately stored in bar-code-labeled polypropylene vials at −75°C (Park et al., 2015). In our laboratory, we run assays for CSF biomarkers, using INNOTEST enzyme-linked immunosorbent assay (ELISA) kits (Fujirebio Europe N.V.) (Jang et al., 2022). We applied CSF Aβ42 levels to CSF Aβ parameters.

### Statistics

Independent student’s *t*-test was used to analyze the continuous variables, and the chi-square test was used for the dichotomous variables.

To determine the cutoff points for plasma Aβ42/40 ratio and CSF Aβ42, respectively, ROC curve analyses were performed using dichotomised Aβ PET status (Aβ PET+/−) as an endpoint. The cutoff points were identified as the value that gave the maximum Youden index (sensitivity + specificity − 1) from this ROC analysis. We defined plasma Aβ42/Aβ40 or CSF Aβ42 as abnormal (plasma+ or CSF+) when those were lower than the cutoff values, respectively. The concordance rates of Aβ PET and fluid Aβ measures were calculated as the number of fluid+/PET+ plus fluid−/PET− cases over the total number of participants in the analysis.

Receiver operating characteristic curves were analyzed to assess factors affecting the predictive accuracy of fluid Aβ biomarkers (plasma Aβ42/40 ratio in Cohort 1 and CSF Aβ42 in Cohort 2) for Aβ PET positivity. Model 1 has plasma Aβ42/40 ratio (Cohort 1) or CSF Aβ42 (Cohort 2) as an only independent variable. Model 2, 3, and 4 include age, cognitive stage (CU vs. CI) or the presence of *APOE* ε4 allele (either heterozygotes or homozygotes), as an additional variable, respectively, and model 5 includes all these four variables. The AUC of multiple models was compared using the DeLong method with Bonferroni correction in Cohort 1 and Cohort 2, respectively.

Statistical analyses were performed using SPSS v.25 (IBM). Statistical significance was set at *p* < 0.05.

## Results

### Characteristics of the participants

Table 1 shows the demographics and clinical characteristics of the participants. In both cohorts, compared to the Aβ PET negative group, the Aβ PET positive group was more likely to carry an *APOE* ε4 allele (*p* < 0.001) and was more likely to have cognitive impairment (*p* < 0.001). However, there were no differences in age, gender, and years of education between the Aβ PET positive and negative groups in both Cohort 1 and Cohort 2.

**Table 1:** Demographic and clinical characteristics of the study participants.

	Cohort 1: Aβ plasma–Aβ PET cohort			Cohort 2: Aβ CSF–Aβ PET cohort		
	Aβ PET(−)	Aβ PET(+)	*p* value	Aβ PET(−)	Aβ PET(+)	*p* value
*N* (%)	243 (49.8%)	245 (50.2%)		58 (26.7%)	159 (73.3%)	
Age, years	69.7 ± 8.5	70.2 ± 9.4	0.473	68.7 ± 9.5	66.5 ± 8.9	0.196
Gender, female	147 (60.5%)	156 (63.7%)	0.469	32 (55.2%)	98 (61.6%)	0.390
Education, years	10.9 ± 5.1	10.9 ± 4.6	0.946	12.5 ± 4.6	11.9 ± 4.3	0.376
*APOE* ε4 carrier	201 (17.3%)	153 (62.4%)	< 0.001	12 (20.7%)	96 (60.4%)	< 0.001
Heterozygotes (ε2/ε4, ε3/ε4)	40	111		12	75	
Homozygotes (ε4/ε4)	3	41		0	21	
Cognitive stage			< 0.001			< 0.001
CU, *N* (%)	131 (53.9%)	17 (6.9%)		14 (24.1%)	6 (3.8%)	
CI (MCI, Dementia), *N* (%)	112 (46.1%)	228 (93.1%)		44 (75.9%)	153 (96.2%)	
Plasma Aβ42/40 (ratio)	0.295 ± 0.061	0.243 ± 0.056	< 0.001			
CSF Aβ42, pg./mL				974.96 ± 379.33	498.56 ± 240.03	< 0.001

Values are represented as mean ± standard deviation or number (%) unless otherwise indicated.

Aβ, β-amyloid; CSF, cerebrospinal fluid; PET, positron emission tomography; SD, standard deviation; APOE, apolipoprotein E; CU, cognitively unimpaired; CI, cognitively impaired.

### Relationships between fluid Aβ biomarkers’ levels and Aβ Pet positivity

**Figure 1:**
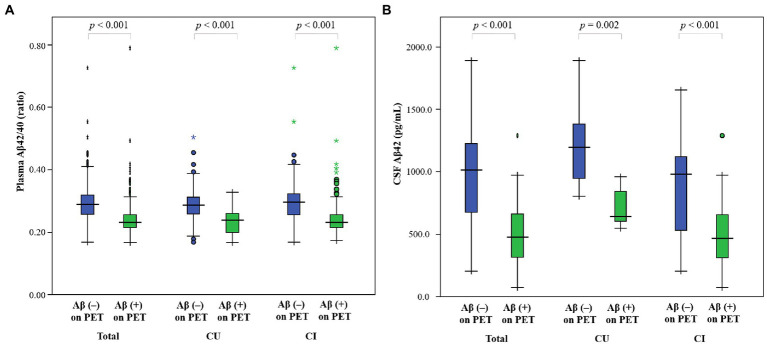
Comparison of fluid Aβ levels according to cognitive stage in **(A)** Cohort 1 (Aβ plasma–Aβ PET cohort) and **(B)** Cohort 2 (Aβ CSF–Aβ PET cohort). Error bars indicate standard errors. The *p* values from comparisons according to Aβ deposition on PET are indicated at the top of each plot. Aβ, β-amyloid; CSF, cerebrospinal fluid; PET, positron emission tomography; CU, cognitively unimpaired; CI, cognitively impaired.

In Cohort 1, the Aβ PET positive group showed significantly lower plasma Aβ42/40 levels than the Aβ PET negative group (*p* < 0.001) whereas in Cohort 2, the Aβ PET positive group showed significantly lower CSF Aβ42 levels than the Aβ PET negative group (both *p* < 0.001; Figure 1).

The plasma Aβ42/Aβ40 cutoff according to the highest Youden index was 0.2576. A good concordance rate between plasma Aβ42/Aβ40 and Aβ PET status was achieved (371/488 = 76.0%). The remaining 117 participants with discordant positivity included 58 (11.9%) plasma+/PET− and 59 (12.1%) plasma−/PET+ participants. On the other hand, the CSF Aβ42 cutoff based on the highest Youden index was 833.33. A concordance rate between CSF Aβ42 and Aβ PET status was high at 85.1% (183/215). The remaining 32 participants with mismatched positives included 14 (6.5%) CSF+/PET– participants and 18 (8.4%) CSF–/PET+ participants. Representative concordant and discordant cases between plasma Aβ42/Aβ40 and Aβ PET and representative concordant and discordant cases between CSF Aβ42 and Aβ PET are shown in Supplementary Figure.

### Clinical information affecting the predictive accuracy of fluid Aβ biomarkers

**Figure 2:**
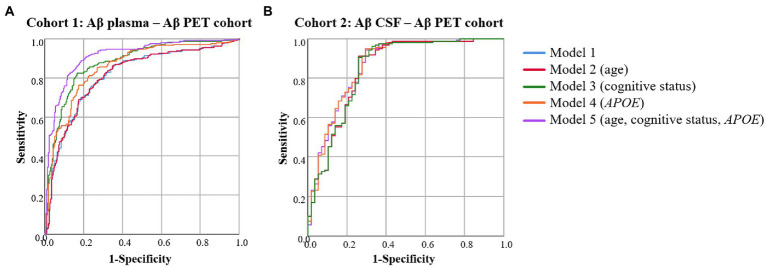
Receiver operating characteristic curves of the models to predict Aβ PET positivity in **(A)** Cohort 1 (Aβ plasma–Aβ PET cohort) and **(B)** Cohort 2 (Aβ CSF–Aβ PET cohort). Aβ, β-amyloid; CSF, cerebrospinal fluid; PET, positron emission tomography; *APOE*, apolipoprotein E.

In Cohort 1 (or Aβ plasma–Aβ PET cohort), the AUC values were 0.814 in model 1, 0.814 in model 2, 0.879 in model 3, 0.858 in model 4 and 0.913 in model 5 (Figure 2A; Table 2A). DeLong tests with Bonferroni correction revealed that the AUC values were significantly increased in models 3, 4, and 5 compared to model 1 (*p* < 0.001, *p* = 0.011, *p* < 0.001, respectively) (Table 2A). That is, when cognitive stage or *APOE* ε4 were added to plasma Aβ, performance of predicting Aβ accumulation was increased.

**Table 2:** The values of area under the curve of all models in (A) Cohort 1 (Aβ plasma – Aβ PET cohort) and (B) Cohort 2 (Aβ CSF – Aβ PET cohort).

(A) Cohort 1: Aβ plasma–Aβ PET cohort	AUC	95% CI	p value (DeLong test)	
Model 1	Plasma Aβ	0.814	0.775–0.853	reference
Model 2	Plasma Aβ + age	0.814	0.775–0.853	1.000
Model 3	Plasma Aβ + cognitive stage	0.879	0.847–0.910	< 0.001
Model 4	Plasma Aβ + *APOE* ε4	0.858	0.824–0.891	0.011
Model 5	Plasma Aβ + age + cognitive stage+ *APOE* ε4	0.913	0.887–0.939	< 0.001
(B) Cohort 2: Aβ CSF–Aβ PET cohort	AUC	95% CI	p value (DeLong test)	
Model 1	CSF Aβ	0.848	0.781–0.915	reference
Model 2	CSF Aβ + age	0.848	0.781–0.914	0.798
Model 3	CSF Aβ + cognitive stage	0.848	0.782–0.916	1.000
Model 4	CSF Aβ + *APOE* ε4	0.866	0.804–0.927	0.542
Model 5	CSF Aβ + age + cognitive stage+ *APOE* ε4	0.867	0.806–0.927	0.460

Aβ, β-amyloid; CSF, cerebrospinal fluid; PET, positron emission tomography; AUC, area under the curve; 95% CI, 95% confidence interval; APOE, apolipoprotein E.

In Cohort 2 (or Aβ CSF–Aβ PET cohort), the AUC values were 0.848 in model 1, 0.848 in model 2, 0.848 in model 3, 0.866 in model 4 and 0.867 in model 5 (Figure 2B; Table 2B). DeLong test with Bonferroni correction showed no significant differences between all the models (Table 2B).

## Discussion

In the present study, we determined whether age, *APOE* genotype, and cognitive stage are affecting the predicting accuracy of plasma Aβ and CSF Aβ for amyloid PET positivity in Aβ plasma–β PET cohort (Cohort 1) and Aβ CSF–Aβ PET cohort (Cohort 2). We found that both CSF Aβ42 and plasma Aβ42/40 biomarkers predicted Aβ PET positivity with high accuracy. More importantly, cognitive stage and *APOE* ε4 genotype increased the predicting accuracy of plasma Aβ42/40 but not the predicting accuracy of CSF Aβ42, for Aβ PET positivity. Therefore, our findings suggest that plasma Aβ42/40 can be a useful predictor of Aβ PET positivity as well as CSF Aβ42, particularly when considered among with clinical information in patients in the AD continuum.

We found that both CSF Aβ42 and plasma Aβ42/40 showed a good AUC for predicting Aβ PET positivity. CSF Aβ and Aβ PET are known to be the most validated biomarkers for reflecting the presence of the soluble and fibrillary forms of Aβ, respectively (Blennow et al., 2015), which were also confirmed by autopsy studies (Strozyk et al., 2003; Clark et al., 2012). CSF Aβ biomarkers have been reported frequently to show highly concordance with Aβ PET status (Janelidze et al., 2016, 2017; Lewczuk et al., 2017; Hansson et al., 2018; Lee et al., 2020), which is consistent with our study finding. Also, our study recapitulates that plasma Aβ, measured by the HPLC-MS/MS method, also predicts Aβ PET status with a high accuracy, in line with earlier studies (Jang et al., 2019; Pascual-Lucas et al., 2021). In our previous study, Jang et al. demonstrated that plasma Aβ42/40 levels were well-correlated with quantitative PET uptake measured by dcCL units (Jang et al., 2021). Thus, our findings support the utility of both CSF Aβ42 and plasma Aβ42/40 as useful biomarkers for predicting Aβ PET status.

The concordance of plasma Aβ42/40 and Aβ PET was 76.0%, which was lower than the concordance of CSF Aβ42 and Aβ PET (85.1%). Our findings are in alignment with those of previous studies showing that the concordance of plasma Aβ42/40 and Aβ PET (76.3–81.5%) (Verberk et al., 2018; Jang et al., 2021; Pascual-Lucas et al., 2023), is lower than that of CSF Aβ and Aβ PET (74.9–92.5%) (Palmqvist et al., 2014; Lewczuk et al., 2017; Mo et al., 2017; Hansson et al., 2018). Previously, plasma Aβ and CSF Aβ detected non-fibrillar soluble Aβ, while Aβ PET measured fibrillar Aβ. Thus, fluid Aβ biomarkers including plasma Aβ and CSF Aβ might represent earlier changes in AD progression than Aβ PET findings, resulting in fluid Aβ biomarkers+/PET– discordant cases (Palmqvist et al., 2016; Schindler et al., 2019; Burnham et al., 2020). However, fluid Aβ biomarkers–/PET+ discordant cases still exist. A longitudinal trajectory study showed that either CSF Aβ or Aβ PET might become abnormal first in different times and might represent different rates of brain Aβ accumulation (Sala et al., 2021). Our previous study also suggested that plasma Aβ42/40 and Aβ PET measures may not be directly interchangeable, but rather reflect independent processes (Jang et al., 2021). Alternatively, the discordant cases of fluid Aβ biomarkers and Aβ PET might be related to the differences in analytical and biological variability. Furthermore, our findings indicating that the discrepancy of plasma Aβ42/40 and Aβ PET seems to be higher than that of CSF Aβ42 and Aβ PET might be related to the differences in the analytical and biological variability between the plasma and CSF measurements.

Our major finding was that *APOE* ε4 genotype increased the predicting accuracy of plasma Aβ42/40 ratio for Aβ PET positivity. Our findings are in line with previous studies showing that adding *APOE* genotype improved the accuracy of plasma Aβ42/40 (Verberk et al., 2018; Janelidze et al., 2021; West et al., 2021). Verberk et al. (2018) showed that AUC value of plasma Aβ42/40 improved when *APOE* genotype was combined, but they did not compare the AUC values between the two models. Other studies revealed an increase in the accuracy of plasma Aβ42/40 for predicting Aβ PET status when combined with other variables including age and *APOE* genotype (Doecke et al., 2020; West et al., 2021; Pascual-Lucas et al., 2023). In another previous study, the AUC value was 0.84 when predicting Aβ PET with plasma Aβ42/40 alone, which increased to 0.88 when *APOE* ε4 genotype was added (Li et al., 2022), in line with our finding. Thus, the predictability of plasma Aβ42/Aβ40 for Aβ PET status might be improved by adding the information of *APOE* genotype, which is easily accessible in clinical practice.

The reason why *APOE* ε4 genotype increased the predicting accuracy of plasma Aβ42/40 for Aβ PET positivity, but not the predicting accuracy of CSF Aβ42, remains unknown. However, our findings suggest that this fact might be related to differences in the physiology of production and clearance between plasma Aβ and CSF Aβ. That is, considering that Aβ in the brain is removed by transportation across the BBB into the venous blood, plasma Aβ levels may depend on the conditions of BBB. Supporting this idea, it has been reported that, the presence of the *APOE* ɛ4 allele affects the loss of BBB integrity through having a toxic effect on CNS endothelial cell tight junctions, eventually resulting in enhanced permeability of the BBB (Marco and Skaper, 2006). These BBB dysfunctions might subsequently cause increased Aβ burdens and Aβ transport failure leading to Alzheimer’s disease and cognitive impairment (Cai et al., 2018). Also, CNS apolipoprotein E protein and Aβ are ligands for low-density lipoprotein receptor-related protein 1 (LRP-1) that is known to be a major transporter of Aβ out of the brain (Monro et al., 2002; Zlokovic, 2004; Donahue and Johanson, 2008). *APOE* ɛ4 may influence the transporter of Aβ at the BBB *via* altering the LRP-1-mediated clearance of soluble Aβ (Fullerton et al., 2001; Wahrle et al., 2007). Thus, plasma Aβ, but not CSF Aβ, would be affected by the presence of the *APOE* ɛ4 allele.

We also found that cognitive stage increased the predicting accuracy of plasma Aβ42/40 ratio for Aβ PET positivity as well. Our findings are in line with another study revealing that the addition of cognitive stage improves the predictive performance for detecting Aβ PET status than the use of plasma Aβ42/40 ratio alone (Jang et al., 2021). Our findings might be explained by the effects of the cognitive stage on BBB dysfunction. That is, the pathological process of Aβ deposition could lead to BBB dysfunction and BBB dysfunction could also cause Aβ production and Aβ transport failure, which becomes a damaging feedback loop and eventually leads to cognitive decline and progression of AD (Bowman et al., 2007, 2018; Cai et al., 2018; Sweeney et al., 2018). Decreased clearance of Aβ from the brain into the blood would also be influenced by alteration of BBB permeability during the Alzheimer’s process (Ramanathan et al., 2015). Therefore, we should consider cognitive stage to predict the accuracy of plasma Aβ42/40 for Aβ PET positivity.

When predicting Aβ PET positivity with CSF Aβ42, the accuracy did not increase even when the variable of *APOE* genotype was combined to CSF Aβ42. These are in line with previous studies in which the presence of *APOE* ɛ4 allele was a non-significant predictor in the model for predicting Aβ PET positivity with CSF Aβ (Lewczuk et al., 2017). There was no significant improvement in the predicting accuracy when other variables such as age, cognitive stage, memory function, and hippocampus volume were added to CSF Aβ, suggesting that the CSF Aβ alone was highly concordant with Aβ PET status and this agreement is independent of the other variables (Palmqvist et al., 2014; Schindler et al., 2019).

The strength of the current study is that the association between fluid Aβ biomarkers levels and Aβ uptakes on PET scans were investigated in a cohort of AD continuum. However, the present study has several limitations. First, we used Aβ PET findings, not autopsy findings that could make a definite diagnosis, to predict Aβ accumulation with plasma Aβ and CSF Aβ. This point, however, might be mitigated by means that Aβ PET status was highly correlated with the post-mortem Aβ burden (Clark et al., 2012). Second, the Aβ plasma–Aβ PET and the Aβ CSF–Aβ PET cohorts were composed of different participants. Further research in participants with all studies including plasma Aβ, CSF Aβ, and Aβ PET is needed. Nevertheless, our study is noteworthy in that we could suggest the potential clinical utility of plasma Aβ biomarker as a predictor for Aβ accumulation in the brain when considered with *APOE* genotype and cognitive stage. Aβ PET is limited by cost and availability in the clinical practice. Also, the determination of CSF Aβ has the problem of invasiveness. Therefore, if we understand the characteristics of plasma Aβ and how its prediction for CNS pathology is affected by other clinical factors, plasma Aβ could be more efficiently used in future clinical practice, as it reflects soluble Aβ, which can be more sensitive to find earlier changes in brain β-amyloidosis (Jang et al., 2021). Finally, the measures of CSF Aβ40 were not available for the present study. Although we have reported the high accuracy of CSF Aβ42 alone to predict Aβ PET positivity in our previous studies (Lee et al., 2020), future studies using CSF Aβ42/40 ratio would be more convincing for the CSF-plasma comparable analysis.

In conclusion, our findings suggest that plasma Aβ42/40 can be a useful predictor of Aβ PET positivity as well as CSF Aβ42, particularly when considered among with clinical information in patients in the AD continuum. The clinical utility of plasma Aβ as useful biomarkers will aid the early detection of AD pathologic changes and the development of prevention or treatment strategies.

## Data availability statement

The raw data supporting the conclusions of this article will be made available by the authors, without undue reservation.

## Ethics statement

The studies involving human participants were reviewed and approved by Institutional Review Board of Samsung Medical Center. The patients/participants provided their written informed consent to participate in this study.

## Author contributions

MC was a major contributor to writing the manuscript and interpreted the data. JG, JA, LS, SC, and MP-L contributed to the methodology and acquisition of data. HK, JK, and DN interpreted the data and revised the manuscript for intellectual content. HJ and SS designed and conceptualized the study, and revised the manuscript for intellectual content. All authors contributed to manuscript revision, read, and approved the submitted version.

## Funding

This research was supported by a grant of the Korea Health Technology R&D Project through the Korea Health Industry Development Institute (KHIDI), funded by the Ministry of Health and Welfare and Ministry of Science and ICT, Republic of Korea (grant number: HU20C0111 and HU22C0170); a grant of the Korean Health Technology R&D Project, Ministry of Health and Welfare, Republic of Korea (HI19C1132); the National Research Foundation of Korea (NRF) grant funded by the Korea government (MSIT) (NRF-2019R1A5A2027340 and NRF-2020R1A2C1009778); Future Medicine 20*30 Project of the Samsung Medical Center [#SMX1230081]; “National Institute of Health” research project (2021-ER1006-02); the Korea Health Technology R&D Project through the Korea Health Industry Development Institute (KHIDI) and Korea Dementia Research Center (KDRC), funded by the Ministry of Health and Welfare and Ministry of Science and ICT, Republic of Korea (HU20C0414); the Korea Health Industry Development Institute (No. HU22C0052); and the DPUK through the Medical Research Council (MR/L023784/2), and partly supported by Institute of Information and communications Technology Planning and Evaluation (IITP) grant funded by the Korea government (MSIT) (No.2021-0-02068, Artificial Intelligence Innovation Hub).

## Conflict of interest

JA, LS, SC, and MP-L are full-time employees of Araclon Biotech-Grifols, the manufacturer of the mass spectrometry test (ABtest-MS).

The remaining authors declare that the research was conducted in the absence of any commercial or financial relationships that could be construed as a potential conflict of interest.

## Publisher’s note

All claims expressed in this article are solely those of the authors and do not necessarily represent those of their affiliated organizations, or those of the publisher, the editors and the reviewers. Any product that may be evaluated in this article, or claim that may be made by its manufacturer, is not guaranteed or endorsed by the publisher.

## Footnotes

^1^ http://www.gaain.org

## Supplementary material

SUPPLEMENTARY FIGURE S1 Examples of concordant and discordant cases in **(A)** Cohort 1 (Aβ plasma – Aβ PET cohort) and **(B)** Cohort 2 (Aβ CSF – Aβ PET cohort). Four representative cases of 18F-futemetamol PET are shown. The Scale bar indicates standardized uptake values. Aβ, β-amyloid; CSF, cerebrospinal fluid; PET, positron emission tomography; dcCL, direct comparison Centiloid.
