# Supplementary figures and images for: Contribution of clinical information to the predictive performance of plasma β-amyloid levels for amyloid positron emission tomography positivity
Source: Front Aging Neurosci. 2023 Mar 14;15:1126799. doi: 10.3389/fnagi.2023.1126799 (PMC10044013; doi:10.3389/fnagi.2023.1126799)

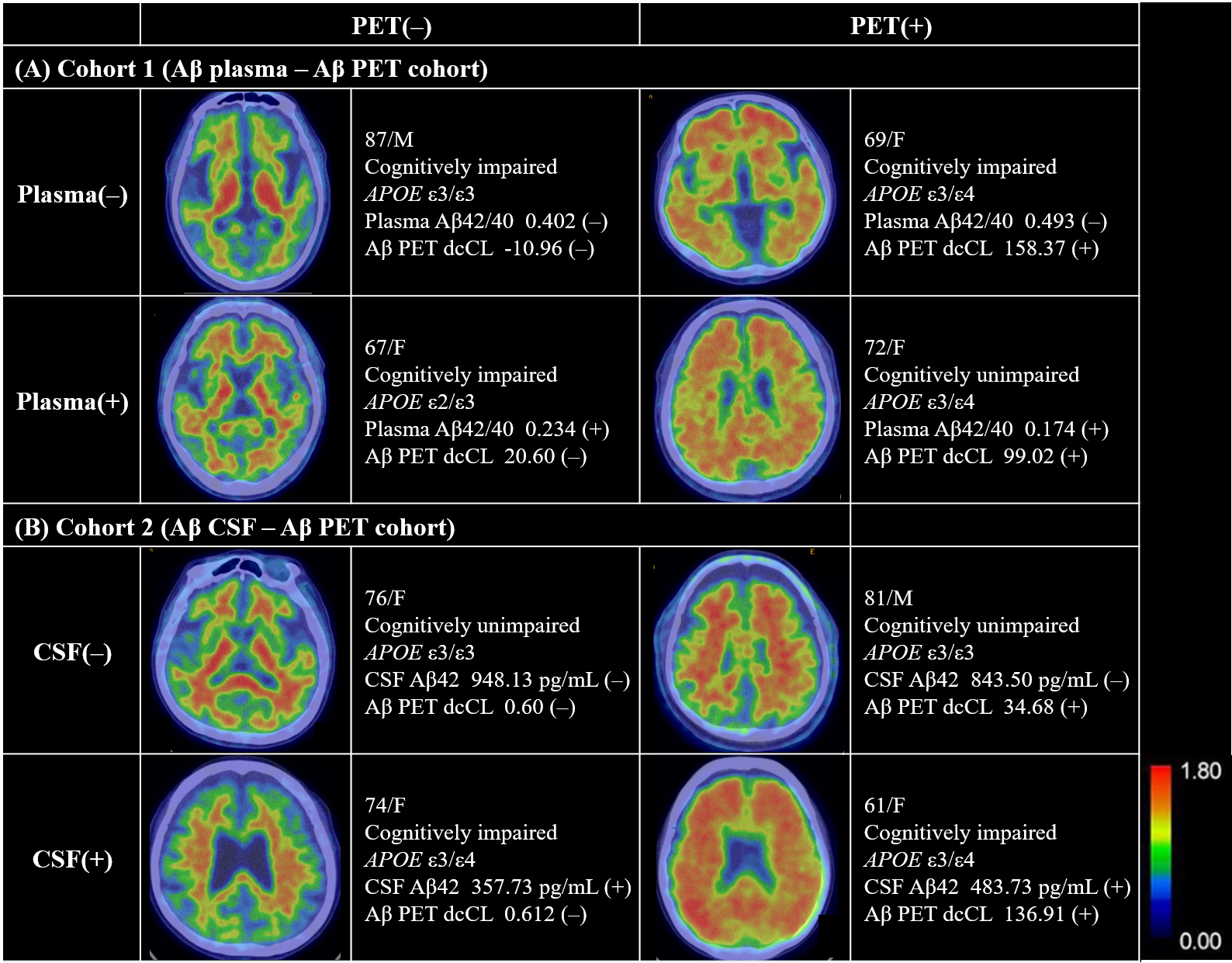

Supplement: SUPPLEMENTARY FIGURE S1 — Examples of concordant and discordant cases in (A) Cohort 1 (Aβ plasma – Aβ PET cohort) and (B) Cohort 2 (Aβ CSF – Aβ PET cohort). Four representative cases of 18F-futemetamol PET are shown. The Scale bar indicates standardized uptake values. Aβ, β-amyloid; CSF, cerebrospinal fluid; PET, positron emission tomography; dcCL, direct comparison Centiloid. [file Image_1.TIF]
